# Integrative Pre-Breeding for Biotic Resistance in Forest Trees

**DOI:** 10.3390/plants10102022

**Published:** 2021-09-26

**Authors:** Melisa Guevara-Escudero, Angy N. Osorio, Andrés J. Cortés

**Affiliations:** 1 Department de Ciencias Forestales, Facultad de Ciencias Agrarias, Universidad Nacional de Colombia, Sede Medellín, Medellín 050034, Colombia; mguevarae@unal.edu.co (M.G.-E.); anosorioh@unal.edu.co (A.N.O.); 2 Main Address: Corporación Colombiana de Investigación Agropecuaria AGROSAVIA, C.I. La Selva, Km 7 Vía Rionegro, Las Palmas, Rionegro 054048, Colombia

**Keywords:** antagonistic biotic interactions, biotic stress, omnigenetics, pre-adaptation, genomics

## Abstract

Climate change is unleashing novel biotic antagonistic interactions for forest trees that may jeopardize populations’ persistence. Therefore, this review article envisions highlighting major opportunities from ecological evolutionary genomics to assist the identification, conservation, and breeding of biotic resistance in forest tree species. Specifically, we first discuss how assessing the genomic architecture of biotic stress resistance enables us to recognize a more polygenic nature for a trait typically regarded Mendelian, an expectation from the Fisherian runaway pathogen–host concerted arms-race evolutionary model. Secondly, we outline innovative pipelines to capture and harness natural tree pre-adaptations to biotic stresses by merging tools from the ecology, phylo-geography, and omnigenetics fields within a predictive breeding platform. Promoting integrative ecological genomic studies promises a better understanding of antagonistic co-evolutionary interactions, as well as more efficient breeding utilization of resistant phenotypes.

## 1. Introduction

Forecasting tree responses to climate change has typically considered shifts in their phenology and geographic distribution in the face of changing abiotic pressures. However, biotic interactions may equally be altered by niche decoupling [1], in turn affecting tree populations’ adaptive and migration potentials [2]. Therefore, better and more integrative predictions require comprehensively assessing whether antagonistic and facilitated biotic interactions may be enhanced, maintained, or decoupled as a result of environmental fluctuations [3,4]. Otherwise, key forest services, both ecological (i.e., resources of biodiversity) and industrial (i.e., renewable materials such as wood, cellulose for the pulp industry, and lignin and hemicelluloses for energy production), may be jeopardized [5].

Fluctuating biotic interactions due to antagonistic biota such as pathogens, insect pests, and weeds are responsible for yield losses ranging from 17.2% up to 30.0% in major food crops [6]. Although studied to a lesser extent, the forestry sector presumably exhibits similar losses [7]. Despite the lack of explicit comprehensive assessments for forest trees, the effect of altered biotic stresses on forests must not be downplayed [8].

The pace at which climatic threats may be altering biotic interactions urges intensifying novel experimental and analytical approaches to better comprehend the effect on the plant disease triangle (PDT). PDT postulates that any plant disease is the result of the interaction between a host’s genotype, the biotic stress, and their environment [9]. Genomic prediction, machine learning, and gene editing strategies, although usually disentangled, offer powerful opportunities for trans-disciplinary and emergent inferences at the interface among the fields of forest genomics, pathology, and ecology [10].

Therefore, this review article aims to highlight major ecological evolutionary genomics’ tools to assist identification, conservation, and pre-breeding of biotic resistance in forest trees. Specifically, our first goal is to summarize the main trends when exploring the genomic basis (i.e., architecture) of biotic stress resistance in forest tree species, by wondering whether resistance types segregate as major Mendelian loci, a prediction from the Fisherian runaway [11] pathogen–host concerted arms-race [12] evolutionary model [13], or exhibit polygenic signatures across various phases of stress perception, signal amplification, and downstream responses. As a second goal, we aim to outline an integrative pipeline to detect and harness natural tree adaptation and pre-breeding for resistance to biotic stresses [14]. Powering integrative studies will enable a better understanding of climate change effects on forest trees’ responses to biotic stresses.

## 2. Mechanisms and Genomic Architecture of Biotic Stress Resistance

### 2.1. Mechanisms of Antagonistic Biotic Interactions

**Figure 1:**
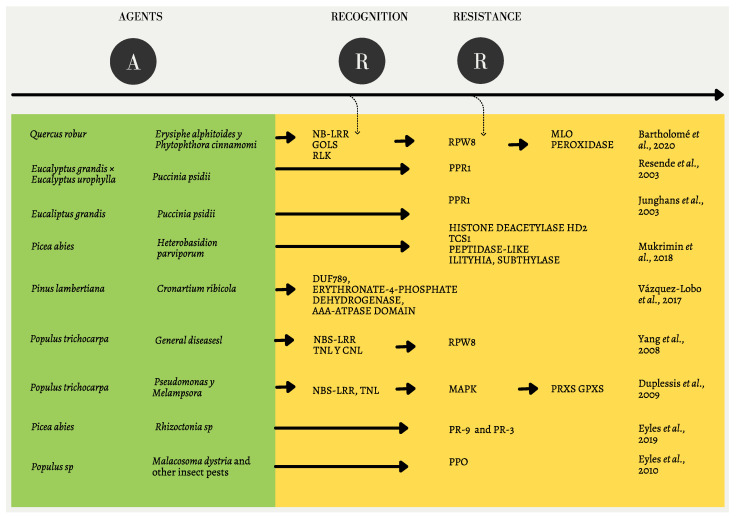
Classification of key genes, proteins, and genomic regions reported to be involved in the biotic resistance across diverse plant species. Agents: plant species and pathogens that interact in an antagonist manner. Recognition: proteins, genes, and genomic regions reported in the recognition phase of pathogens by the host species. Resistance: proteins, genes, and genomic regions capable to confer resistance from the host plat species to the pathogen. For bibliographic details, refer to Table S1. Figure and Table S1 are illustrative, and do not mean to be exhaustive [16,17,18,19,20,21,22,23].

The molecular mechanisms of antagonistic biotic interactions can be synthesized in three steps: attack, recognition phase, and resistance responses (Figure 1). A series of favorable environmental conditions enable a pathogen attack, triggering plant recognition through the interaction between resistance genes and small-secreted pathogens called effectors. This interaction induces effector-activated immunity (ETI) that can modulate plant cell physiology, or even elude the plant defense response [15].

After the recognition phase, plants will usually activate their defense responses, which can be induced in multiple signaling pathways through invader-specific effectors. Ultimately, plants deploy a variety of morphological and physiological mechanisms to reduce damage from pests and pathogens such as the production of toxic or antimicrobial chemicals or proteins, programmed cell death, and compensation [14].

### 2.2. Genomic Architecture of Biotic Stress Resistance

When summarizing the gemomic architecture of resistance to biotic stress in forest tree species, it is outstanding to find a similar proportion of articles classifying the resistance trait as Mendelian, as well as polygenic (Table 1). This invites us to rethink the Fisherian runaway [11] arms-race pathogen–host model of concerted evolution [13], classically perceived as the null hypothesis [12]. The validity of this null premise is likely scale-dependent, being mostly applicable at a macro-scale (i.e., when studying upstream genes and markers associated with the resistant phenotype). At a more fine downstream scale, biotic stress resistance traits may tend to exhibit polygenic architectures as part of the omnigenic model [24], in which the regulatory networks of genes are sufficiently interconnected so that there are core regulatory genes linked with pleiotropic ones [24].

### 2.3. Broad Responses to Antagonistic Biotic Interactions

Genes and markers involved in resistance to biotic stress can be divided into two categories. The first is involved in pathogen recognition (e.g., RGA genes, resistance gene analogues), while the other is more involved in defense response *per se* (e.g., DGA genes or defense gene analogues) [30]. The best-studied RGAs are leucine-rich repeats of nucleotide binding sites, kinase receptor-like proteins, pentatricopeptide repeats, and apoplastic peroxidases. Nucleotide-binding site leucine-rich repeats (NBS-LRRs) contain a nucleotide-binding site (NBS), and leucine-rich repeats (LRRs). They function as intracellular immunoreceptors that recognize, directly or indirectly, pathogenic effectors specifically encoded by the avirulence gene [31]. The LRR motif is responsible for recognition specificity, and is often involved in protein–protein interactions [21].

TNL and CNL proteins appear within this category, which recognize pathogen effectors that are secreted into the cell, allowing plants to trigger the effector-activated immune response (ETI). This response generally results in the production of ITP/ITN, calcium, phytohormones, burst of oxidative reactive oxygen species, activation of the MAPK cascade, and transcription of defense genes of hypersensitive response to limit pathogen spread [32].

Meanwhile, TM-LRRs can be subdivided into two classes: receptor-like kinases (RLKs), and other receptor-like proteins (RLPs). RLKs and RLPs are pattern recognition receptors (PRRs) for a wide range of pathogens. They are used as the first line of recognition by plants and for the immune response triggered by microbial inducers PAMP or MAMP, which are highly conserved microorganisms’ structural molecules such as flagellin, chitin, and lipopolysaccharides [33]. RLKs and RLPs are structurally similar proteins. The former are involved in plant development and cell surface defense, deployment of plant receptors to detect pathogens, and signal translation through activated signaling pathways to trigger innate immune responses [16]. It should be noted that not all RLPs are involved in disease resistance, some may play a role in plant development [32]. For its part, the ATPase domain AAA has been associated with cell death and hypersensitive responses in plants, suggesting that it could be involved in biotic stress responses. Similarly, the erythronate-4-phosphate protein has been associated with vitamin B6 and in the defense of *Pinus lambertiana* Dougl. [20].

Another group of genes involved in plant resistance mechanisms are the pathogen response or defense genes (DGA genes), which are not as conserved as the RGA (and thus are more likely to exhibit a polygenic tendency [30]). However, there are genes associated with specific pathogens, as is the case of RPW8, PPR1, MAPK, and PPO, as reported in several studies [17,18,21,22,23]. In 2018, it was shown that an ILYTHIA-like protein (ILA) might be involved in the regulation of ROS accumulation and programmed cell death in response to pathogen attack. Furthermore, it is known to be important in induced defense reactions in *Arabidopsis* [19]. Of course, further sub-classifications exist, more downstream given the functionality of these two categories, although often ambiguous owing to rampant pleiotropy.

Qualitative and quantitative resistances are two other classifications that are recurrent when discussing responses to antagonistic biotic interactions given various degrees of complexity in the genomic architecture of the resistant phenotype [34]. The former classification deals mostly with major genes [35] (i.e., directly derived from the Mendelian paradigm). The latter typically involves multiple genes with minor effects (i.e., as from the Fisherian infinitesimal [11] polygenic model [36]). R genes are the classical example of qualitative resistance [37], generally conferring complete resistance to a specific pathogen, and thus are the most-easy to manipulate through modern gene editing approaches [38].

Qualitative resistance has been detected mainly in the defense of plants against biotrophic pathogens, while quantitative resistance is more often involved in the defense response to broader plant pathogens, from biotrophs, hemibiotrophs, and necrotrophs. For instance, the Pto gene in tomato confers qualitative resistance, similar to RPS2 in *Arabidopsis* and N gene (mosaic virus resistance gene) in tobacco [32]. Quantitative resistance is controlled by multiple genes, each contributing to partial resistance [39].

Overall, reconciling alternative classifications of the molecular responses to biotic stresses is challenging owing to reiterative knowledge gaps on the functionality or the nature of the associated genomic regions. Besides, rampant polymorphism at downstream levels [40], as well as the broad mosaic of antagonistic biotic genetic interactions in the resistance pathway at such scales, impose further research bottlenecks. Still, this extensive polymorphism at the downstream components is what ultimately enables the diversity of plant responses to biotic stresses, and offers sufficient standing genetic pre-adaptations, in addition to paralogous’ sub-functionalization, in order to cope with evolving pathogen entities.

## 3. Major Challenges When Studying Tree Defense Responses to Biotic Stresses

The stability of forest biomass in the world is threatened by the incidence of pests and diseases that cause a continuous deterioration of health [41]. It is clear that the study of the genetic evolution of both hosts and pathogens is a promising resource. However, it is not yet feasible to breed for resistant phenotypes in the reiterative absence of basic information for many species. Available reports tend to focus on temperate and boreal tree species, leaving aside tropical and subtropical forests. Only 33% of the surveyed studies are located in tropical regions (Table 1) [42]. Additionally, studies give priority to herbaceous species (of short generation times) owing to the complexity of the anatomy of forest species, and the time effort required to study perennials [14].

Therefore, it is necessary to improve data compilation and access on plant genetic resources [43] with a forestry perspective. This implies strengthening joint field trials, systematic characterization of diverse genetic materials, and quality control at nurseries, while developing technical protocols for the elaboration of inventories and reinforcing information systems in such a way that they become broadly accessible [44]. Advances and discoveries of resistance genes in forest trees are undeniably increasing, yet there is still a lack of data convergence and free accessibility [15].

Unfortunately, studies of genetic biotic resistance in forest stands still lack cohesion. Although the infectious agent is known in all surveyed studies (Table 1), the regulatory processes behind, and the key proteins involved during the attack and in the development of the disease, are often undetected. Furthermore, in some cases, the genetic mapping resolution is limited to entire genomic clustered hotspots [31] and chromosomes, making a precise reconstruction of the complex genetic basis unfeasible.

Sustainable forestry development must also better integrate disciplines that adjust to the particularities of each locality. In this way, it may be achievable to select elite genotypes, and identify genetically improved clones with narrow pre-adaptations [45] that can overcome the biotic adversities they face [46]. Since the XIX century, the need to harness alternatives for the conservation [47] of global tree diversity has arisen, recognizing the management of tropical forests as a key element [14]. Therefore, ecological, biogeographical, and genetic disciplines must be integrated in order to better understand changing antagonistic biotic interactions [48], while slowing down populations’ extirpation [49]. Such trans-disciplinary approaches at the interface of evolutionary and ecological genetics [50] may ultimately serve to explore whether the phylogenetic basis of resistance is enhanced by ecological plasticity [51] and adaptation [52] in highly variable regions [53], often understudied from a forest pathology perspective [16].

## 4. Novel Strategies to Speed up Tree Pre-Breeding for Biotic Stress Resistance

**Figure 2:**
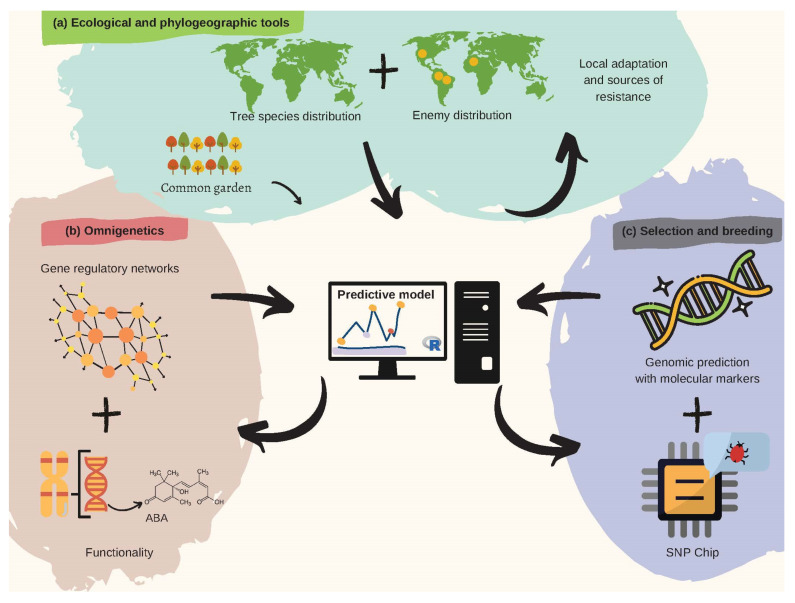
Recommendations for harnessing pre-breeding in forest tree pathology. (**a**) Spatial modeling of plant species and overlapping infectious agents is useful to identify resistance hotspots and pre-adaptations, later validated by common garden trials. (**b**) Gene regulatory networks (i.e., omnigenetics) enable to better explain the missing heritability in biotic resistance traits (diagram above within the pink bubble), while understanding trans-generational epigenetic inheritance as well as the metabolic bases for resistance (diagram below within the pink bubble). The diagram above functionality refers to a hypothetical example on metabolic pathways (e.g., biosynthesis of abscisic acid). (**c**) A last step aiming to search for molecular markers involved in attack and defense processes can further leverage pre-breeding for forest pathology via integrative predictive modeling of polygenic breeding values (diagram above within the purple bubble), which can easily be scalable using targeted genotyping SNP arrays/chips (diagram below within the purple bubble).

Tree pre-breeding requires an interdisciplinary approach [54] to speed up breeding cycles and increase selection accuracy in the face of jeopardizing climate change effects [55]. Such intersection (Figure 2) must happen among pathologists, botanists, ecologists, ecophysiologists, geneticists, biometeorologists, dendrologists, paleoecologists, and phylogeographers. DNA variation studies must not be disconnected from ecologically relevant trait variation in provenance trials, capable of revealing pre-adaptations to naturally high incidence of pathogenic fungi in humid niches, and insects that threaten the diversity of trees worldwide [56].

### 4.1. Leveraging Integrative Approaches

In recent years, there has been increasing interest in merging several disciplines to gather more cohesive inferences in the field of forest genetics [57]. For instance, molecular genetics has successfully coupled with the field of ecological biogeography and landscape ecology [58] to better understand the adaptive equilibrium between tree populations, and the niches where they occur [59]. This innovation has in turn informed potential long-term evolutionary responses [60].

The impact of pests and diseases on the distribution of forest trees, and their regeneration mechanisms [61], has been suggested as one of the main explanations for the high diversity of species in tropical forests [48]. Consequently, this hotspot is a valuable reservoir for sources of resistance. A better interaction between the fields of tree pre-breeding and phylogeography will allow a more comprehensive reconstruction of the ecological drivers, including antagonistic biotic interactions, of today’s diversity [7]. A first step in this regard requires mapping the geographical distributions of genealogical lineages across heterogeneous landscapes, for both pests and hosts (Figure 2a). The extent of overlap among trees and pathogens’ geographical distributions can then be used as a proxy to infer the likelihood for concerted evolution [62], and potential pre-adaptations [63]. Within this framework, evolutionary genetics may also offer promising avenues because it specifically deals with the mechanisms that explain the existence and maintenance of genetic variation across traits. While joint species distribution modeling prospectively informs the magnitude of biotic interactions at regional scales [48], multi-locality common garden (i.e., provenance trials), and clonal trials with trans-located biotic treatments, can in turn illuminate the other end of the spectrum [15]. Controlled reciprocal trials may offer a more mechanistic understanding of the ecological genomics of co-evolutionary interactions at local-scales [64].

### 4.2. Acknowledging and Harnessing Local Adaptation in Biotic Interactions

Local adaptation conceptualizes the trend that local populations tend to have a higher average fitness in their native environment than in other environments, or when compared with foreign introduced populations [65]. Despite this, local adaptation [66] has typically been over-simplified as driven by abiotic [67] heterogenetic interactions [68]. Until now, biotic interactions are starting to be recognized as major drivers, too [69]. Yet, one of the key challenges that remain in the study of local adaptation is to explore its genomic basis [48], either via loci exhibiting antagonistic pleiotropy or conditional neutrality [70]. These ecological and phylogeographic trends must be interpreted in the light of interacting gene networks (i.e., omnigenetics approach [24], Figure 2b).

Attempts to identify candidate genes of adaptive importance, and to relate genetic variation in these genes to phenotypic expressions in multi-locality field trials, have typically been hampered by a complex polygenic architecture [71], and a limited understanding of the physiological trade-offs [48]. For instance, theoretical expectations dictate that local selection at a single locus will promote local adaptation in the absence of gene flow (i.e., selection–migration balance [65]). However, more complex polygenic quantitative adaptation can even be established and maintained in the presence of high gene flow [72]. While the discipline of molecular quantitative tree genetics [73] merges with the field of ‘big data’ analytics [74], an expanded view of complex traits is arising, moving from a polygenic framework to a view in which all genes are liable to affect adaptation to biotic stresses (the omnigenic model described above, Figure 2b [24]), so that most heritability can be explained by the effects of rare variants, their second order epistatic interactions, and with epigenetic factors, even accounting in this way for transgenerational epigenetic inheritance [75]. However, looking back, the metabolic basis of tree evolution still has the potential to improve plantations’ yields because natural selection has tested more options than humans ever will. Mining the molecular footprint of selection and adaptation from in situ sampling for tree pre-breeding and climate adaptation will benefit from bridging the gap between phenotyping and genotyping across provenances, and the more deterministic quantitative and population genetic models.

A useful type of polygenic model, yet to be calibrated within an omnigenic framework [24], is genomic prediction (GP) [76]. Predictive breeding via GP allows assessing genetic estimated values (GEVs) for biotic stress resistance [77]. GP uses historical resistance data to calibrate marker-based infinitesimal additive predictive models [78], which provide a more comprehensive representation of a quantitative polygenic trait than traditional genetic mapping [79], a tendency that several biotic resistances have started exhibiting [80]. Therefore, GP offers a key path to assist the introgression breeding of biotic stress resistance from key donors (via genomic-assisted recurrent backcrosses—GABC, as successfully applied in the breeding program for blight resistant in American chestnut trees [81]). GP’s predictive ability can be significantly enhanced after performing *a priori* weighted resistance mapping through more conventional methods such as quantitative-trait loci (QTL) mapping or genome-wide association studies (GWAS) [82]. These mapping strategies enable choosing target SNP arrays for high throughput genotyping of multi-parental populations [83] via SNP-Chips (Figure 2c).

GP may also go beyond pre-breeding efforts, and feedback on restoration optimization [84] and provenance characterization [85] (e.g., by predicting biotic resistance and yield) even across thousands of half-sib families that could hardly be tested at once in field and lab trials for pests and herbivore resistance [86]. Expectations within these half-sib families are likely similar to the ones previously discussed, which are as follows: (i) a nascent trend towards a more polygenetic architecture of the resistance, and (ii) the occurrence of pleiotropic genes in response to multiple biotic stresses despite the apparent absence of phenotypic correlations in the components of resistance [77].

### 4.3. Genetic Edition Coupled with Gene Drives May Enable Tree Defense Responses

Genetic drift refers to random allelic fluctuations within genepools [87]. It is typically a consequence of limited population size and rampant selection, and thus becomes stronger in secluded hosts and pathogens’ populations. Rare alleles are likely to disappear completely from populations, while previously polymorphic loci might become fixed. Remarkably, in some cases, pathogens may overcome natural genetic drift by utilizing genetic elements from their host as a way to develop resistance to plant defenses. For instance, whitefly, through a horizontal gene transfer event, acquired the plant-derived phenolic glycoside malonyltransferase gene (BtPMaT1), which allows whiteflies to neutralize phenolic glycosides [88].

On the other hand, modern CRISPR/Cas9 gene editing technology is capable of modifying the immune response function in eukaryotic cells via a highly specific RNA-guided complex [89]. This technology has broad applications in all biological fields, including tree pathology [90]. Bottlenecks are the availability of fine-mapped candidate genes for resistance with major effects, in vitro protocols for tissue culture, and legal regulation [91]. Still, the prospect for gene editing remains open. Interestingly, coupling gene editing with selfish elements in Mendelian segregation distortion due to meiotic drive [92] may efficiently introgress resistance at the population level [93] in a snowball manner [94]. Although promising, combining gene editing with gene drives remains speculative because factual trajectories may prove undesired.

### 4.4. Harnessing Data Access

Joint research efforts to study more systematically the genomics of forest pathology across the enviromics continuum must be envisioned [95]. At the genomic and breeding level, similar initiatives already exist, such as the European EVOLTREE consortium (http://www.evoltree.eu/, accessed on 16 September 2021), and North Carolina State University’s Central America and Mexico Coniferous Resources Cooperative (CAMCORE, https://camcore.cnr.ncsu.edu/, accessed on 16 September 2021) a not-for-profit international tree breeding organization partnered with private companies in the forestry sector around the world. Both alliances may serve as inspiration to build a stronger networking around breeding for biotic resistance in forest trees. Ultimately, promoting more of these partnership efforts will enhance multi-locality trials and data sharing among countries [96], while improving the understanding of the dynamics of co-evolutionary antagonistic interactions in forest ecosystems through genomic, ecological, and evolutionary studies.

Meanwhile, these trans-disciplinary initiatives undeniably require support from both the public (e.g., governmental organizations, country forest agencies, research institutes, and universities) and the private (e.g., forest industries, non-governmental agencies, and downstream manufacturers) sectors. Public–private alliances must be conceived as opportunities to put in place more realistic agreements of data embargo, and for the quarantine of genotypes across institutional and political borders. Unfortunately, overwhelming regulatory issues still tend to put cross-country field trials on hold by preventing an effective mobilization of seeds, seedlings, and data [97]. Hence, multi-lateral strategies must envision enabling data sharing to better foresee regional and continental antagonistic biotic interactions that put forests at risk.

## 5. Conclusions

- Forest pathology must start integrating more thoroughly disciplines that allow understanding the biology and natural evolution of trees under biotic stress, seeking the conservation of the mechanisms by which species have defended themselves from biotic antagonistic agents.
- Polygenetic biotic resistance must be acknowledged as an equally plausible pre-adaptation as Mendelian inheritance. The latter configures a long-standing expectation from the Fisherian runaway pathogen–host concerted arms-race evolutionary model. According to this theoretical paradigm, loci conferring resistance are predicted to evolve in concert due to strong selection towards more durable and unbreakable resistance. In turn, concerted molecular evolution would likely promote long-term linkage disequilibrium and reduced recombination, making several resistance loci behave as a single Mendelian locus.
- Another prerogative must focus on deepening our ecological understating at the pathogen–species–environment interface, while better integrating this classical PDT paradigm with the modern disciplines of forest genomics, molecular biology, phylo-geography, and predictive breeding (i.e., genomic prediction).
- Promoting open access and information agreements among national and international parties (i.e., research centers, tree breeding cooperatives, and industries form the forestry sector) is equally relevant to build more cohesive input datasets to ultimately leverage these ‘big data’ integrative approaches for forest pathology breeding.

## Figures and Tables

**Table 1:** Classification of the genomic architecture of resistance to biotic stress (*s.l.*) in tree species. The genomic architecture is classified as Mendelian (i.e., involving few genomic regions under concerted evolution, as predicted from the Fisherian runaway arms-race pathogen–host model) or polygenic (i.e., involving several loci with moderate/low effects and in linkage equilibrium among them, in absence of genetic hitchhiking). For bibliographic details, refer to Table S1.

Species	Location of Mapping Population	Genetic Markers	Number of Associated Genetic Markers	Genomic Architecture	Ref.
*Quercus robur*	France (bouran y champenoux)	SNPs	2 regions, 165 and 196 genes, respectively	Polygenic	[16]
*Eucalyptus globulus*	Tasmania	AFLPs y SSRs	2 QTLs	Mendelian	[25]
*Eucalyptus grandis × Eucalyptus urophylla*	Brazil	SNPs	1 gen with 218 SNPs	Mendelian	[17]
*Eucaliptus grandis*	Brazil	RAPDs & 1 gen	6 markers, 1 gen	Mendelian	[18]
*Picea abies*	Finland	SNPs	10 SNPs in 8 genes	Mendelian	[19]
*Pinus lambertiana*	North America	SNPs	4 SNPs in 3 genes	Polygenic	[20]
*Populus trichocarpa*	NA	SNPs	NA	Polygenic	[21]
*Populus deltoides*	North Central United States	RAPDs (OPG10 340 y OPZ19 1800)	NA	Polygenic	[26]
*Hevea* spp.	South América	Kruskal–Wallis marker	6 QTLs	Polygenic	[27]
*Eucalyptus*	NA	SSRs, AFLPs, RAPDs, RFLPs, SNPs	1 gen	Mendelian	[28]
*Populus deltoides × Populus trichocarpa*	Europe	RFLPs, RAPDs, AFLPs, STS, SSRs	NA	Polygenic	[29]

## Data Availability

This review does not report novel data. Bibliographic compilation is reported below, as well as in Table S1.

## Supplementary Materials

The following are available online at https://www.mdpi.com/article/10.3390/plants10102022/s1, Table S1: Proteins, genes, and chromosomes involved in the interaction of plant species with various infectious agents. This table provides a list of items that give an overview of the proteins and chromosomes that are involved in both the attack and defense phases of tree species in the presence of a natural biotic enemy. Key observations are highlighted, specifically concerning the functionality of the genes and chromosomes reported by each study. For a graphical representation of this table please refer to Figure 1.
